# Prostate Cancer Diagnosis and Treatment in Elderly Patients: A Cross-Sectional Survey Exploring Practice Patterns and Preferences of Uro-Oncologists in Northeast Italy

**DOI:** 10.3390/diagnostics15091100

**Published:** 2025-04-25

**Authors:** Filippo Carletti, Giuseppe Reitano, Laura Evangelista, Filippo Alongi, Alessandro Antonelli, Umberto Basso, Roberto Bortolus, Matteo Brunelli, Orazio Caffo, Fabrizio Dal Moro, Rocco De Vivo, Mario Gardi, Rossano Girometti, Andrea Guttilla, Fabio Matrone, Matteo Salgarello, Marco Andrea Signor, Fabio Zattoni, Gianluca Giannarini, on behalf of Gruppo Uro-Oncologico del Nord Est (GUONE)

**Affiliations:** 1 Urology Clinic, Department of Surgery Oncology and Gastroenterology, University of Padua, 35122 Padua, Italy; 2 Nuclear Medicine Unit, IRCCS Humanitas Research Hospital, 20089 Milan, Italy; 3 Advanced Radiation Oncology Department, IRCCS Sacro Cuore Don Calabria Hospital, Cancer Care Center, Negrar, 37024 Verona, Italy; 4 Department of Medical and Surgical Specialties, Radiological Sciences and Public Health, Università di Brescia, 25121 Brescia, Italy; 5 Urology Unit, University of Verona, 37129 Verona, Italy; 6 Oncology Unit, Department of Oncology, Istituto Oncologico Veneto IOV-IRCCS, 35128 Padua, Italy; 7 Radiation Oncology Department, Centro di Riferimento Oncologico di Aviano (CRO) IRCCS, 33081 Aviano, Italy; 8 Pathology Unit, University of Verona, 37129 Verona, Italy; 9 Oncology Unit, Santa Chiara Hospital, 38122 Trento, Italy; 10 Department of Oncology, Ospedale san Bartolo, 36100 Vicenza, Italy; 11 Department of Medicine, University of Udine, 33100 Udine, Italy; 12 University Hospital S. Maria della Misericordia, ASUFC, 33100 Udine, Italy; 13 Urology Unit, ULSS 6 Euganea, 35012 Camposampiero, Italy; 14 Nuclear Medicine Unit, IRCCS Ospedale Sacro Cuore Don Calabria, 37024 Negrar, Italy; 15 Radiation Therapy Unit, SMM University Hospital Udine, 33100 Udine, Italy; 16 Urology Unit, SMM University Hospital Udine, 33100 Udine, Italy

**Keywords:** elderly, PCa, prostate cancer, survey, old, aging population

## Abstract

**Background**: The optimal diagnostic and therapeutic strategies for prostate cancer (PCa) in patients aged ≥75 years (mild-old and oldest-old) are still contentious. Resource allocation and ideal treatment for older patients are challenges, mainly due to their comorbidities and reduced life expectancy. This survey aims to assess current clinical practices and the experiences of healthcare providers in the diagnosis and management of elderly patients with PCa. **Materials and Methods:** In Northeast Italy, members of the Gruppo Uro-Oncologico del Nord-Est (GUONE) conducted a survey involving 104 physicians of different specialties (Nuclear Medicine, Medical Oncology, Radiation Oncology, Radiology, Urology) between 1 November 2024 and 30 November 2024. The survey encompassed 51 questions, evaluating various diagnostic and therapeutic scenarios. **Results:** Digital rectal exam (DRE) was recommended by 35.9% of physicians for patients aged 75 or older at risk of PCa. PSA testing was continued in 76.3% of these patients. For 36.5% of the physicians, there should be no age limit for prostate biopsy. Moreover, 42.6% of physicians recommended a magnetic resonance imaging (MRI)-guided prostate biopsy regardless of age. A prostate biopsy was deemed mandatory before initiating any form of hormonal therapy by 57.7% of the participants. For 22.3% and 34.7% of physicians, there should be no age limit for prostate MRI and PET/CT for staging purposes. Interestingly, PET/CT was not recommended in 52% of cases as a staging tool for patients older than 85 years. For patients without comorbidities, the age limit to consider radical prostatectomy (RP) was 75, with 58.6% of physicians in favor. There were no definitive limits for radiotherapy (RT). Chemotherapy had an age limit for 81.6% of the respondents; for 18.4%, 22.5%, and 26.5% of physicians, age limits were 75, 80, and 85 years, respectively. The use of androgen receptor pathway inhibitors (ARPIs) had no definitive age limits for 46.5% of respondents. For patients with no comorbidities and low-volume metastatic PCa, the preferred option was androgen deprivation therapy + ARPIs + RT. The follow-up schedule after RP or RT exhibited heterogeneity with no consensus regarding the frequency of PSA testing or the age at which it should be discontinued. **Conclusions:** This survey highlights the need for consensus guidelines in diagnosing and managing mild-old and oldest-old elderly PCa patients. With the aging population, standardized protocols are essential to ensure optimal care.

## 1. Introduction

The increasing age of the global population poses significant challenges for the management of prostate cancer (PCa) [1], particularly given that most PCa diagnoses occur in men over 65 years, with a significant proportion over 75 [2,3]. Elderly patients often encounter delays in diagnosis, limited access to oncology care, and may face hesitation in receiving immediate curative treatment [4]. This is due to concerns about life expectancy, comorbidities, polypharmacy, financial strain, and social factors affecting caregiver responsibility [5], which can result in either undertreatment or overtreatment. Age-based guidelines for PCa treatment are limited in scope; additionally, elderly patients are often underrepresented in clinical trials [6], leaving gaps in evidence-based guidelines that specifically address their needs. The first International Society of Geriatric Oncology (SIOG) guidelines in 2010 emphasized that PCa treatment decisions should rely on health status, not chronological age, using comprehensive geriatric assessment (CGA) tools to guide care [7]; in the 2014 update, the G8 screening tool was introduced for identifying patients who may benefit from further geriatric evaluation [8]. This approach was fully endorsed in 2017 by the European Association of Urology with the new EAU/ESTRO/SIOG guidelines [9,10,11]. Chronological age fails to reflect the biological diversity among elderly patients [12], whose health statuses vary widely; treatment decisions should be made according to patient’s preferences and based on the individual health status, rather than solely relying on their age [13]. In practice, despite the critical importance of conducting a geriatric assessment to optimize patient care plans, these tools remain underutilized due to limited awareness and insufficient familiarity among healthcare providers [14]. In an ESMO/SIOG survey on the management of older patients with cancer, only 66% of responders were aware of the existence of the G8 screening tool, and only 52% used it in their clinical practice [14,15]. While molecular imaging and novel therapeutic approaches offer potential benefits, they add layers of complexity to clinical decision-making, as elderly patients often face an increased risk of adverse effects, including cardiovascular complications and cognitive impairment linked to hormone therapies. Despite notable progress, inconsistencies in professional guidelines regarding age-based screening and treatment thresholds still contribute to uncertainty in managing PCain elderly patients. The absence of a standardized protocol emphasizes the compelling need for guidelines that consider essential factors such as functional status, cognitive ability, life expectancy, and patient preferences. These elements are critical in allowing clinicians to personalize treatment strategies without sacrificing the importance of quality of life [16]. This study seeks to address these complexities by surveying a multidisciplinary team of experienced specialists to identify common practices and decision-making patterns in managing elderly with suspected or biopsy-proven PCa and to clarify areas of consensus and divergence in current PCa management, contributing to the development of evidence-based guidelines that support balanced, patient-centered PCa care.

## 2. Materials and Methods

This survey was conducted by members of Gruppo Uro-Oncologico del Nord Est (GUONE) group from 1 November 2024 to 30 November 2024, targeting experienced physicians (EP) practicing in Northeast Italy across multiple specialties, including Nuclear Medicine, Medical Oncology, Radiation Oncology, Radiology, and Urology. All participants provided informed consent. The survey instrument was developed following a comprehensive review of existing guidelines and expert feedback from a multidisciplinary panel. After pilot testing, the final questionnaire consisted of 51 items covering diagnostic and treatment practices, follow-up strategies, and age-specific considerations. Questions were structured around clinical scenarios designed to explore how age, comorbidities, and functional status influence clinical decisions in elderly PCa management. To address the heterogeneity within the elderly population, age was categorized into the following subgroups: youngest-old (70–74 years), middle-old (75–85 years), and oldest-old (>85 years). This stratification was incorporated to elucidate differences in clinical decision-making across these subpopulations, acknowledging that older age groups, particularly the middle-old and oldest-old, represent the primary focus of this investigation. These distinctions align with established classifications in the geriatric literature and underscore the unique clinical profiles of older cohorts, particularly in the context of PCa management. The final questionnaire comprised 51 items addressing demographic and practice characteristics, diagnostic approaches including digital rectal exam (DRE) and PSA testing protocols, biopsy practices, imaging protocols focusing on magnetic resonance imaging (MRI) and positron emission tomography (PET)/computed tomography (CT) utilization, treatment selection criteria, follow-up strategies, and age-specific considerations in management decisions. The survey was distributed via e-mail through Google Forms to ensure standardized data collection. To maximize response rates, two reminder emails were sent at weekly intervals. The electronic platform incorporated logic checks to ensure data quality and completeness throughout the collection process. Descriptive statistics, including frequencies and percentages, were obtained for all survey items, by using Excel.

## 3. Results

### 3.1. Demographic Characteristics of Responders

One hundred and four EP participated in the survey. Urologists constituted approximately half of the respondents (49%), followed by oncologists (26%), radiation oncologists (14%), radiologists (4%), nuclear medicine physicians (3%), and other (3%). Most respondents (90%) indicated that PCa was their primary area of interest. The median years of professional experience was 7 (IQR: 2–19). Regarding the annual patient load, 75% reported managing ≥50 PCa cases annually, whereas 25% treated <50 such cases. Geographical distribution revealed that most respondents were based in Veneto (69%), followed by Friuli-Venezia Giulia (27%), Trentino-Alto Adige (2%), Lombardia (1%), and Emilia Romagna (1%).

### 3.2. Digital Rectal Examination and PSA Testing

**Figure 1:**
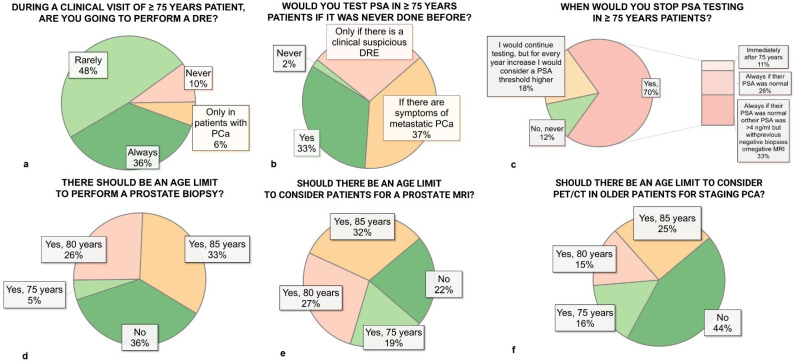
Differences among responders. DRE = digital rectal examination; PSA = prostate-specific antigen; MRI = magnetic resonance imaging; PET/CT = positron emission tomography/computer tomography; PCa = prostate cancer. (**a**) Frequency of digital rectal examination (DRE) during clinical visits in patients aged ≥75 years. (**b**) Attitude toward prostate-specific antigen (PSA) testing in patients ≥ 75 years old who have never been tested before. (**c**) Criteria and timing for stopping PSA testing in patients ≥ 75 years old. (**d**) Age limit to perform prostate biopsy. (**e**) Age limit to consider patients for prostate magnetic resonance imaging (MRI). (**f**) Age limit to consider PET/CT for staging prostate cancer (PCa) in older patients.

Regarding diagnostic practices, 48% of respondents reported rarely performing a digital rectal examination (DRE) in patients aged 75 years or older, while 36% always performed it (Figure 1a). For PSA testing in patients aged 75 years or older who had not previously been tested, 37% would test only if symptoms of metastatic disease were present, 33% would test regardless of symptoms, and 28% would test only if positive DRE (Figure 1b). When it came to discontinuing PSA monitoring in 75 ≥ years patients, 70% of physicians would stop testing. Among these, 33% would discontinue PSA monitoring only for patients with a history of normal PSA levels and previous negative biopsy or negative MRI; 26% of respondents would cease testing only in patients with consistently normal PSA results, while 11% would discontinue testing immediately. Furthermore, 18% would raise PSA thresholds with age, and 12% indicated they would never stop PSA testing. (Figure 1c).

### 3.3. Prostate Biopsy

Prostate biopsy practices also showed significant heterogeneity. While 36% of physicians supported no absolute age limit for prostate biopsy, others set limits at 70 years (5%), 80 years (26%), and 85 years (33%) (Figure 1d). Preferred biopsy techniques for patients aged 75 years or older were evenly divided between transrectal ultrasound-guided (50%) and transperineal approaches (50%). MRI-guided biopsy recommendations also varied: 42% recommended it regardless of age, 44% recommended it only for clinically significant prostate cancer, and 14% never recommended it. Notably, 58% of respondents considered prostate biopsy mandatory before initiating any treatment, while 38% considered PSA values and the risk of clinically significant prostate cancer (csPCA) before deciding to possibly avoid biopsy.

### 3.4. Imaging

Imaging practices revealed age-related restrictions among physicians. For prostate MRI, 22% supported no age limit, while others set age limits at 75 years (19%), 80 years (27%), or 85 years (32%) (Figure 1e). PET/CT for staging exhibited similar patterns: 44% supported no age limit, but the remaining 56% defined limits at 75 years (16%), 80 years (15%), or 85 years (25%). For restaging purposes, 44% supported no age limit, with others setting limits at 75 years (16%), 80 years (15%), or 85 years (15%) (Figure 1f).

### 3.5. Life Expectancy and Comorbidity Assessment Tools

When estimating life expectancy in ≥70 years patients with PCa, responders predominantly relied on general performance status indices, including the Eastern Cooperative Oncology Group (ECOG) Performance Status (58%) and the Karnofsky Performance Status (54%) and Charlson Comorbidity Index (CCI) (50%), often choosing two or more indices simultaneously. For evaluating comorbidities in patients aged 70 years and older, the use of prostate cancer-specific tools, such as the Prostate Cancer-specific Comorbidity Index (13%) and the G8 Geriatric Screening Tool (3%), were notably less common compared to the broader use of the CCI (65%) and ECOG (52%).

### 3.6. Treatment Options

**Figure 2:**
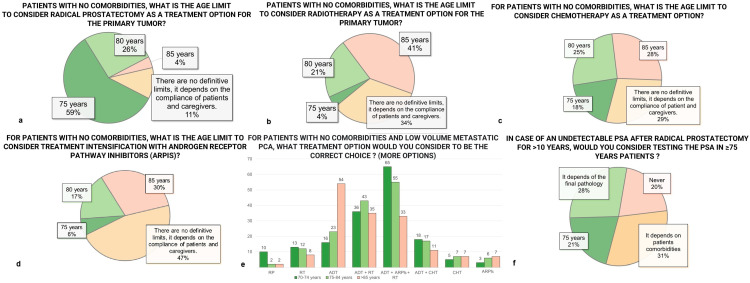
Differences among responders; PCa = prostate cancer; PSA = prostate-specific antigen; RP = radical prostatectomy; RT = radiotherapy; ADT = androgen deprivation therapy. (**a**) Suggested upper age limits for considering radical prostatectomy as a treatment option for the primary tumor. (**b**) Suggested upper age limits for considering radiotherapy as a treatment option for the primary tumor. (**c**) Suggested upper age limits for considering chemotherapy as a treatment option. (**d**) Attitudes toward age thresholds for treatment intensification with androgen receptor pathway inhibitors (ARPIs). (**e**) Preferred treatment strategies in patients with no comorbidities and low-volume metastatic PCa, stratified by age groups. (**f**) Opinions on whether PSA testing should be continued in men aged ≥75 years with undetectable PSA more than 10 years after radical prostatectomy.

Treatment approaches showed clear age-related trends. For surgical management in patients without comorbidities, 59% favored radical prostatectomy (RP) up to 75 years, with support decreasing at older ages (Figure 2a). Radiotherapy (RT) recommendations were more evenly distributed: 34% reported no definitive age limit but based decisions on patient or caregiver compliance, while age limits of 75, 80, and 85 years were set by 4%, 21%, and 41%, respectively (Figure 2b).

### 3.7. Systemic Therapy

Systemic therapy practices also varied. Chemotherapy age limits were set by 71% of respondents, with specific thresholds at 75 years (18%), 80 years (25%), and 85 years (27%) (Figure 2c). Androgen receptor pathway inhibitors (ARPIs) showed more flexibility, with 467% supporting no definitive age limits, 6% setting the threshold at 75 years, 17% at 80 years, and 30% at 85 years (Figure 2d). For patients with no comorbidities and low-volume metastatic prostate cancer, the preferred treatment across all age groups (70–74, 75–85, >85 years) was the combination of androgen deprivation therapy (ADT), ARPIs, and RT (Figure 2e).

### 3.8. Follow-Up

Follow-up protocols also showed significant variation. Among patients aged 75 or older who had undergone RP and maintained undetectable PSA levels for over 10 years, 31% of respondents based PSA monitoring on patient comorbidities, 29% on final pathology, and 20% either always or never continued monitoring. A similar trend was observed post-radiotherapy, where 35% based follow-up on comorbidities, 25% on final pathology, and 20% either always or never continued monitoring.

### 3.9. Clinical Trial Participation

Guidelines for clinical trial participation differed by intervention type. For new radiotherapy modalities, 26% set no age limit, while 36% set the limit at 75 years. Participation in drug trials showed similar trends, with 27% setting no age limit and nearly equal proportions setting limits at 80 years (30%) or 85 years (29%).

### 3.10. Communication Practices

Communication practices highlighted a thoughtful approach to patient factors. Moreover, 33% of physicians always disclosed a prostate cancer diagnosis to patients aged 75 years or older, and 62% tailored their approach based on patients’ cognitive and functional status. When discussing the prognosis of metastatic disease, functional status played an even more significant role, with 68% of EP making disclosure decisions based on cognitive and functional status, while 18% adhered to a policy of universal disclosure.

## 4. Discussion

As the global population ages [17], the proportion of men over the age of 75 is increasing. In the near future, a growing percentage of these individuals is expected to have a life expectancy exceeding 10 years [18], presenting new challenges in prostate cancer (PCa) management. This survey reveals substantial variability in management approaches for elderly PCa patients, particularly in decisions surrounding PSA testing, biopsy, and imaging. These decisions often reflect a delicate balance between life expectancy and quality of life, highlighting the pressing need for clearer, evidence-based guidelines tailored to elderly populations. Currently, no comprehensive guideline exists to address the complexities of PCa in elderly patients. Our findings emphasize the necessity of developing such protocols to optimize healthcare spending, address resource allocation challenges between elderly and younger populations, and ensure equitable access to care. The diversity in responses further illustrates a misalignment between current clinical guidelines and real-world practices. Key areas of variability identified in this survey include the following:

**PSA Testing:** Our findings revealed that most physicians would discontinue PSA monitoring in elderly patients under specific circumstances, such as consistently normal results, negative imaging, or biopsy findings. However, the literature provides conflicting recommendations on PSA screening in this population [19,20], particularly regarding its utility in improving outcomes versus the risks of overtreatment [21,22]. Guidelines from the major organizations, including the American Urological Association (AUA), the US National Comprehensive Cancer Network (NCCN), the American Cancer Society, and the European Association of Urology (EAU-EANM-ESTRO-ESUR-SIOG) offer varying and sometimes contradictory recommendations, further complicating clinical decision-making in elderly individuals [23].

**Imaging:** There was significant variability in imaging practices, including prostate MRI and PET/CT. In this study, prostate MRI was recommended for older patients more liberally than the current literature suggests, which primarily focuses on younger populations [24]. Similarly, PET/CT was often employed for staging and restaging, with age restrictions varying widely, some respondents applying no age limits, while others set restrictions based on advanced age. Given the high costs and limited availability of molecular imaging, its use in elderly patients should be critically evaluated, particularly when the benefit is uncertain [25]. Current cost-effectiveness models for PCa management primarily target populations under 70 years, leaving a significant gap in evidence for older individuals [26,27]. On the other hand, the risk of drug interactions and toxicity is very low, particularly with newer radiotracers like prostate-specific membrane antigen (PSMA). Additionally, molecular imaging provides the ability to target therapies to specific sites, potentially avoiding unnecessary treatment in cases of recurrence when results are negative and follow-up is recommended [28]. These findings highlight the compelling need for age-specific imaging guidelines and further research to assess the cost-effectiveness of advanced imaging modalities in elderly PCa patients.

**Comprehensive Geriatric Assessment:** Although tools for geriatric assessment are widely recommended for elderly patients, only a small minority of respondents reported consistently using them. Instead, reliance was primarily on broader indices like the CCI and the ECOG PS, often without a clear preference for a specific tool. When asked, EP typically indicated the use of more than one tool. This suggests that although there is recognition of the value of these assessments, practical barriers, such as time constraints and lack of training, prevent the widespread adoption of tailored geriatric assessment tools [14,15].

**Treatment Options:** While many respondents favored radical treatments, such as prostatectomy and radiotherapy up to a certain age, the literature increasingly emphasizes the importance of tailoring interventions to individual patient profiles [29,30,31,32].

This study has limitations that impact the generalizability of its findings. The sample size was insufficient to allow robust stratification by specialty or years of experience, limiting how these factors might influence clinical decision-making. The survey’s geographic focus on northeastern Italy may not fully represent practices in other regions or healthcare systems. For example, the use of certain drugs (such as ARTA) is exclusive to oncologists in northern Italy but not in the south, which may reflect potential differences in management approaches. Selection bias is also possible, as physicians with a specific interest in geriatric oncology may have been more likely to participate.

This survey highlights the compelling need for consensus guidelines to standardize the diagnosis and management of prostate cancer (PCa) in elderly patients. As the population ages, developing evidence-based protocols becomes increasingly essential to provide optimal and patient-centered care for this growing demographic. Currently, a lack of standardized practices leads to considerable variability, which can result in both undertreatment and overtreatment, compromising the quality of care and increasing strain on healthcare systems.

Consensus guidelines that incorporate factors such as life expectancy, functional status, and patient preferences may enable clinicians to balance quality of life with therapeutic efficacy. Future research should focus on the cost-effectiveness of PCa management for elderly patients to facilitate broader, evidence-based use of available tools in clinical practice.

## 5. Conclusions

Creating standardized protocols specifically designed for PCa management in the elderly is essential to address the unique needs of this population, improve clinical outcomes, and support healthcare systems in delivering effective and resource-efficient care. By addressing these gaps, healthcare systems can enhance care quality and optimize resource allocation for an aging global population.

## Data Availability

The original contributions presented in the study are included in the article, further inquiries can be directed to the corresponding author.

## Abbreviations

The following abbreviations are used in this manuscript:

PCa	Prostate cancer
ARPIs	Androgen receptor pathway inhibitors
CGA	comprehensive geriatric assessment
SIOG	International Society of Geriatric Oncology
DRE	Digital Rectal examination
ADT	Androgen Deprivation Therapy
RT	Radiotherapy
RP	Radical Prostatectomy
EP	Experienced Physicians

## References

[1] James N.D., Tannock I., N’Dow J., Feng F., Gillessen S., Ali S.A., Trujillo B., Al-Lazikani B., Attard G., Bray F. (2024). The Lancet Commission on prostate cancer: Planning for the surge in cases. Lancet.

[2] Rawla P. (2019). Epidemiology of Prostate Cancer. World J. Oncol..

[3] Vatandoust S., Kichenadasse G., O’Callaghan M., Vincent A.D., Kopsaftis T., Walsh S., Borg M., Karapetis C.S., Moretti K. (2018). Localised prostate cancer in elderly men aged 80–89 years, findings from a population-based registry. BJU Int..

[4] Bratt O., Folkvaljon Y., Eriksson M.H., Akre O., Carlsson S., Drevin L., Lissbrant I.F., Makarov D., Loeb S., Stattin P. (2015). Undertreatment of Men in Their Seventies with High-risk Nonmetastatic Prostate Cancer. Eur. Urol..

[5] Graham L.S., Lin J.K., Lage D.E., Kessler E.R., Parikh R.B., Morgans A.K. (2023). Management of Prostate Cancer in Older Adults. Am. Soc. Clin. Oncol. Educ. B.

[6] Bin Riaz I., Islam M., Ikram W., Naqvi S.A.A., Maqsood H., Saleem Y., Riaz A., Ravi P., Wang Z., Hussain S.A. (2023). Disparities in the Inclusion of Racial and Ethnic Minority Groups and Older Adults in Prostate Cancer Clinical Trials: A Meta-analysis. JAMA Oncol..

[7] Droz J.-P., Balducci L., Bolla M., Emberton M., Fitzpatrick J.M., Joniau S., Kattan M.W., Monfardini S., Moul J.W., Naeim A. (2010). Background for the proposal of SIOG guidelines for the management of prostate cancer in senior adults. Crit. Rev. Oncol. Hematol..

[8] Droz J.-P., Aapro M., Balducci L., Boyle H., Broeck T.V.D., Cathcart P., Dickinson L., Efstathiou E., Emberton M., Fitzpatrick J.M. (2014). Management of prostate cancer in older patients: Updated recommendations of a working group of the International Society of Geriatric Oncology. Lancet Oncol..

[9] Mottet N., Bellmunt J., Briers E., Bolla M., Bourke L., Cornford P., De Santis M., Henry A.M., Joniau S., Lam T.B. (2017). EAU Guidelines on Prostate Cancer 2017.

[10] Cornford P., van den Bergh R.C.N., Briers E., Van den Broeck T., Brunckhorst O., Darraugh J., Eberli D., De Meerleer G., De Santis M., Farolfi A. (2024). EAU-EANM-ESTRO-ESUR-ISUP-SIOG Guidelines on Prostate Cancer—2024 Update. Part I: Screening, Diagnosis, and Local Treatment with Curative Intent. Eur. Urol..

[11] Tilki D., van den Bergh R.C.N., Briers E., Van den Broeck T., Brunckhorst O., Darraugh J., Eberli D., De Meerleer G., De Santis M., Farolfi A. (2024). EAU-EANM-ESTRO-ESUR-ISUP-SIOG Guidelines on Prostate Cancer. Part II—2024 Update: Treatment of Relapsing and Metastatic Prostate Cancer. Eur. Urol..

[12] Droz J.P., Boyle H., Albrand G., Mottet N., Puts M. (2017). Role of Geriatric Oncologists in Optimizing Care of Urological Oncology Patients. Eur. Urol. Focus..

[13] Boyle H., Alibhai S., Decoster L., Efstathiou E., Fizazi K., Mottet N., Oudard S., Payne H., Prentice M., Puts M. (2019). Updated recommendations of the International Society of Geriatric Oncology on prostate cancer management in older patients. Eur. J. Cancer.

[14] Dale W., Williams G.R., MacKenzie A.R., Soto-Perez-De-Celis E., Maggiore R.J., Merrill J.K., Katta S., Smith K.T., Klepin H.D. (2021). How Is Geriatric Assessment Used in Clinical Practice for Older Adults With Cancer? A Survey of Cancer Providers by the American Society of Clinical Oncology. JCO Oncol. Pract..

[15] Baldini C., Brain E.G.C., Rostoft S., Biganzoli L., Goede V., Kanesvaran R., Quoix E., Steer C., Papamichael D., Wildiers H. (2021). 1827P European Society for Medical Oncology (ESMO)/International Society of Geriatric Oncology (SIOG) Joint Working Group (WG) survey on management of older patients with cancer. Ann. Oncol..

[16] Cherny N.I., de Vries E.G., Dafni U., Garrett-Mayer E., McKernin S.E., Piccart M., Latino N.J., Douillard J.-Y., Schnipper L.E., Somerfield M.R. (2017). ESMO-Magnitude of Clinical Benefit Scale version 1.1. Ann. Oncol. Off. J. Eur. Soc. Med. Oncol..

[17] GBD 2015 Mortality and Causes of Death Collaborators (2016). Global, regional, and national life expectancy, all-cause mortality, and cause-specific mortality for 249 causes of death, 1980–2015: A systematic analysis for the Global Burden of Disease Study 2015. Lancet.

[18] CDCP (2003). Trends in Aging—United States and Worldwide. Morb. Mortal Wkly. Rep..

[19] Schaeffer E.M., Carter H.B., Kettermann A., Loeb S., Ferrucci L., Landis P., Trock B.J., Metter E.J. (2009). Prostate Specific Antigen Testing Among the Elderly—When To Stop?. J. Urol..

[20] de Vos I.I., Remmers S., Hogenhout R., Roobol M.J. (2024). Prostate Cancer Mortality Among Elderly Men After Discontinuing Organised Screening: Long-term Results from the European Randomized Study of Screening for Prostate Cancer Rotterdam. Eur. Urol..

[21] Carlsson S.V., de Carvalho T.M., Roobol M.J., Hugosson J., Auvinen A., Kwiatkowski M., Villers A., Zappa M., Nelen V., Páez A. (2016). Estimating the harms and benefits of prostate cancer screening as used in common practice versus recommended good practice: A microsimulation screening analysis. Cancer.

[22] Kensler K.H., Mao J., Davuluri M. (2024). Frequency of Guideline-Discordant Prostate Cancer Screening Among Older Males. JAMA Netw. Open.

[23] Carlsson S.V., Oh W.K. (2024). How Can Guidelines Give Clearer Guidance on Prostate Cancer Screening?. JAMA Oncol..

[24] Drost F.-J.H., Osses D., Nieboer D., Bangma C.H., Steyerberg E.W., Roobol M.J., Schoots I.G. (2020). Prostate Magnetic Resonance Imaging, with or Without Magnetic Resonance Imaging-targeted Biopsy, and Systematic Biopsy for Detecting Prostate Cancer: A Cochrane Systematic Review and Meta-analysis. Eur. Urol..

[25] Krausewitz P., Borkowetz A., Ortner G., Kornienko K., Wenzel M., Westhoff N. (2024). Do we need MRI in all biopsy naïve patients? A multicenter cohort analysis. World J. Urol..

[26] Song R., Jeet V., Sharma R., Hoyle M., Parkinson B. (2022). Cost-Effectiveness Analysis of Prostate-Specific Membrane Antigen (PSMA) Positron Emission Tomography/Computed Tomography (PET/CT) for the Primary Staging of Prostate Cancer in Australia. Pharmacoeconomics.

[27] van der Sar E.C.A., Keusters W.R., van Kalmthout L.W.M., Braat A.J.A.T., de Keizer B., Frederix G.W.J., Kooistra A., Lavalaye J., Lam M.G.E.H., van Melick H.H.E. (2022). Cost-effectiveness of the implementation of [^68^Ga]Ga-PSMA-11 PET/CT at initial prostate cancer staging. Insights Imaging.

[28] Prior J.O., Gillessen S., Wirth M., Dale W., Aapro M., Oyen W.J.G. (2017). Radiopharmaceuticals in the elderly cancer patient: Practical considerations, with a focus on prostate cancer therapy: A position paper from the International Society of Geriatric Oncology Task Force. Eur. J. Cancer.

[29] Mandel P., Chandrasekar T., Chun F.K., Huland H., Tilki D. (2017). Radical prostatectomy in patients aged 75 years or older: Review of the literature. Asian J. Androl..

[30] Dell’Oglio P., Zaffuto E., Boehm K., Trudeau V., Larcher A., Tian Z., Moschini M., Shariat S., Graefen M., Saad F. (2017). Long-term survival of patients aged 80 years or older treated with radical prostatectomy for prostate cancer. Eur. J. Surg. Oncol..

[31] Marotte D., Chand-Fouche M.E., Boulahssass R., Hannoun-Levi J.M. (2022). Irradiation of localized prostate cancer in the elderly: A systematic literature review. Clin. Transl. Radiat. Oncol..

[32] Lundberg F.E., Robinson D., Bratt O., Fallara G., Lambe M., Johansson A.L.V. (2024). Time trends in the use of curative treatment in men 70 years and older with nonmetastatic prostate cancer. Acta Oncol..

